# Influence of FFF Substrate Material and 3D-Printed Surface Morphology on the Formation of Electrodeposited Copper Layers

**DOI:** 10.3390/ma19194040

**Published:** 2026-09-22

**Authors:** Dagmar Klichová, František Botko, Kristyna Sternadelova, Hana Krupova, Svetlana Radchenko

**Affiliations:** 1 Faculty of Manufacturing Technologies, Technical University of Košice, 080 01 Prešov, Slovakia; 2 Faculty of Mechanical Engineering, VSB-Technical University of Ostrava, 70800 Ostrava, Czech Republic

**Keywords:** electroplating, copper coating, polymer composite, surface morphology, non-destructive surface analysis

## Abstract

**Highlights:**

• Substrate material and initial FFF topography influence the morphology of electrodeposited Cu coatings.
• Identical electrodeposition conditions produce different Cu coating morphologies on different FFF substrates.
• Cu deposition modifies peak and valley regions and can locally bridge deeper surface depressions.
• 3D surface topography provides a non-destructive approach for characterising the surface morphology of electrodeposited Cu coatings.

**Abstract:**

Electrodeposition of metallic coatings represents a promising approach for extending the functional properties of additively manufactured polymer components. However, the resulting coating morphology may be influenced not only by the deposition conditions but also by the material and initial topography of the polymer substrate. In this study, the formation of electrodeposited copper (Cu) layers was investigated on Fused Filament Fabrication (FFF) substrates made of polylactic acid (PLA), PLA containing carbon fibres (PLA-CF), PLA containing brass particles (PLA-Brass), and polyethylene terephthalate glycol-modified (PETG), with different initial surface morphologies generated using three printing strategies. All samples received a conductive graphite interlayer and were subsequently electroplated under identical electrochemical conditions. The resulting Cu layers were characterised using cross-sectional observations and non-destructive 3D surface topography analysis. The results showed that the Cu coating retained the basic morphological character of the FFF substrate; however, its resulting topography was not a simple geometric replica of the original surface. Differences among the investigated materials and initial surface topographies remained evident despite identical deposition conditions. Cu layer formation was associated with changes in the peak and valley regions of the surface and local bridging of deeper depressions. The results demonstrate the importance of both substrate material and initial FFF surface topography in determining the resulting morphology of electrodeposited Cu coatings and indicate the potential of 3D surface topography analysis for non-destructive characterisation of Cu surface morphology.

## 1. Introduction

Additive manufacturing based on material extrusion enables the efficient production of lightweight polymer components with complex geometries and a high degree of design flexibility. Fused Filament Fabrication (FFF) is one of the most widely used technologies within this group and enables the processing of not only conventional thermoplastics but also polymer composites containing fibrous or particulate fillers. The properties of FFF components are governed by the combined effects of material composition and process parameters, which influence both their internal structure and the resulting surface characteristics [1].

Metallization represents one approach to extending the functional applications of polymer components. A metallic coating can combine the advantages of lightweight and geometrically versatile polymer substrates with the properties of metals, particularly high electrical and thermal conductivity, improved surface durability, and a metallic appearance. Common metallization methods include physical deposition, electroless plating, and electrolytic metal deposition. Copper (Cu) is particularly attractive because of its high electrical and thermal conductivity and the well-established electrochemistry of Cu deposition [2]. Electroplating of 3D-printed polymer structures has been successfully employed for both selective metallization and enhancement of the electrical conductivity of functional elements and electronic structures [3,4,5].

A fundamental limitation of the direct electroplating of most polymers is their electrical non-conductivity. Therefore, prior to electrodeposition, a conductive surface must be created, or a material containing electrically conductive particles must be applied. Previous studies have demonstrated several possible approaches, including the use of conductive composite filaments, exposure of metallic or carbon particles embedded in the polymer matrix, electroless formation of a conductive base layer, and application of a conductive interlayer [3,4,5,6,7]. These studies also indicate that the characteristics of the resulting Cu layer are governed not only by the electrolyte composition and electrodeposition parameters but also by the properties of the substrate and the characteristics of the surface on which deposition is initiated [4,6,7]. When a conductive interlayer is applied, the influence of the underlying substrate must be interpreted with caution, since the properties of the conductive layer may also affect subsequent Cu deposition.

This aspect is particularly important for additively manufactured polymer substrates because their surfaces exhibit a characteristic topography generated during the layer-by-layer manufacturing process. The initial surface topography may influence nucleation, local growth, and the subsequent development of the metallic layer. Eßbach et al. demonstrated that changes in Fused Deposition Modelling (FDM) surface topology significantly affect the homogeneity and reproducibility of electroplated coatings on Acrylonitrile Butadiene Styrene (ABS) [6]. Krupova et al. investigated the electrolytic deposition of Cu on polyamide 12 (PA12) substrates produced by selective laser sintering (SLS) with different initial surface topographies generated by tumbling and vaporisation. Their results showed that the initial topography influenced the subsequently formed metallic layer; smoother vaporised surfaces resulted in a more uniform Cu layer and did not exhibit the globular growths observed on the tumbled samples. The authors also reported that the electrodeposited layer reproduced the topography of the polymer substrate [8]. The vaporised samples also exhibited lower variability in Cu coating thickness than the samples with rougher surfaces [8]. Ludergnani et al. further demonstrated the influence of FFF structure geometry on Cu penetration and the resulting interconnection between the metallic and polymer components [9], while Melentiev et al., in their study of metallised FDM-ABS, highlighted the importance of the hierarchical morphology of the polymer–Cu interface [10]. These findings indicate that the initial topography of the polymer substrate is one of the factors governing the morphology of the subsequently formed metallic layer.

In addition to surface topography, the material composition of the substrate may also play an important role. Composite filaments containing carbon or metallic fillers exhibit physical properties and surface characteristics that differ from those of unfilled polymers. Lazarus et al. showed that different types of particles in thermoplastic composites can act as nucleation sites for subsequent Cu deposition [7], whereas Sharifi et al. demonstrated the direct metallization of a Cu-PLA composite after exposing copper particles at its surface [11]. Salazar et al. further reported that different geometric arrangements of the surface of a conductive FFF material resulted in differences in Cu film homogeneity and local dendritic growth under identical electroplating conditions [12]. The resulting Cu layer may therefore be influenced by both the material characteristics of the substrate and its initial surface morphology. However, because the substrates in the present study were coated with the same conductive graphite interlayer before electrodeposition, differences in the resulting Cu morphology cannot be attributed solely to the bulk substrate material. This limitation must therefore be considered when evaluating the effect of substrate material.

Our previous work [13] also addressed the influence of the initial surface on the electrolytic deposition of Cu. In that study, PA12 samples produced by SLS and modified before plating by tumbling or vaporisation were evaluated. It was demonstrated that changes in the initial surface morphology were reflected in the topography and characteristics of the subsequently formed Cu layer. The present study extends this research to material extrusion and to polymer and polymer-composite substrates with different material compositions. In contrast to the previous approach, differences in the initial surface topography are not generated by subsequent surface treatment but arise directly during sample fabrication. Recent studies also confirm the growing importance of copper electroplating for the integration of metallic functional elements into Material Extrusion (MEX)/FFF polymer structures [14]. However, systematic comparisons of differences in material composition and initial FFF substrate topography affecting the resulting Cu layer under identical electrodeposition conditions remain limited.

Previous studies have demonstrated that both FFF surface geometry and material composition can influence subsequent metal deposition. However, the relative importance of these two factors for the final topography of an electrodeposited Cu layer has not been sufficiently clarified. The present study therefore combines four FFF substrate materials with three printing strategies under identical graphite-layer preparation and Cu electrodeposition conditions. This experimental design enables the relative influence of substrate material and printing strategy on the resulting Cu surface topography to be quantitatively compared. The primary objective is to establish whether the final Cu surface is dictated more by the underlying substrate or by the initial morphology introduced by the FFF technique.

This comparison is particularly relevant when the electrodeposited Cu layer retains the main morphological features of the original FFF surface rather than completely levelling them. Under these conditions, the initial FFF surface topography remains an important factor influencing the resulting Cu surface. The deposited layer may modify individual peaks and valleys, while the main morphological characteristics of the original FFF surface remain preserved. Consequently, the printing strategy used to generate the original FFF surface may remain an important factor controlling the topography of the subsequently deposited Cu layer.

To assess these changes, it is appropriate to combine destructive cross-section evaluation with non-destructive 3D surface analysis. In this work, topographic analysis is not an objective in itself but serves as a tool for capturing the morphological development of the Cu layer and for searching for parameters that could be used in the future for non-destructive evaluation of metallised polymer components. Areal height parameters provide information about the amplitude of irregularities, while the parameter Sdr (developed interfacial area ratio) appears to be a useful addition, since it also responds to changes in the structure and actual surface area [4,14]. Incorporating volume parameters completes this intricate analysis. These are analysed from the S-F surface without removing the long-wave component with an L-filter, in order to preserve the spatial characteristics related to the real morphology of the FFF surface and the subsequent growth of the Cu layer [14]. If the main topographic characteristics of the original FFF surface remain reflected in the Cu-coated surface, non-destructive 3D topography can provide quantitative information on the morphology of the deposited Cu layer. In this context, similarity in selected height parameters and preservation of the main surface features can be used to assess how the initial FFF topography is transferred to the Cu surface. This approach is limited to the characterisation of surface morphology and does not provide direct information on coating adhesion, microstructure, or functional properties.

The primary objective of the presented research is to evaluate the effects of FFF substrate composition and initial surface morphology, resulting from various printing strategies, on the formation and characteristics of an electrodeposited Cu layer under uniform deposition conditions. To achieve this, PLA, PLA-CF, PLA-Brass, and PETG substrates are compared, each fabricated using three distinct surface generation techniques. The development of the Cu layer is evaluated using cross-sections and non-destructive 3D surface analysis, including height, hybrid and volume parameters. The main objective is to quantitatively compare the relative influence of substrate material and printing strategy on the resulting Cu surface topography and to determine which of these factors has the greater effect. At the same time, the study evaluates the extent to which the initial FFF surface morphology is preserved after Cu deposition and whether 3D surface topography can therefore be used as a non-destructive method for characterising the morphology of the deposited Cu layer.

## 2. Materials and Methods

### 2.1. Materials

Four commercially available filament materials from AURAPOL (Znojmo, Czech Republic) were selected for the experimental study: PLA, PLA-CF, PLA-Brass and PETG. The selected materials allow the comparison of an unfilled polymer substrate, polymer composites with carbon and metal fillers, and a polymer with a different chemical composition. Maintaining the same electrolytic copper plating conditions enables evaluation of the influence of the substrate material composition on the formation and resulting morphology of the Cu layer.

PLA (polylactic acid) was used as a reference unfilled polymer material. Due to the absence of particulate or fibrous filler, the resulting surface is mainly formed by the geometry of individual extruded paths and their mutual overlap. PLA thus provides a reference system for assessing the influence of the printing strategy and for subsequent comparison with PLA-based composite materials.

PLA-CF is a composite material with a PLA matrix containing carbon fibres. The presence of carbon reinforcement distinguishes this material from unfilled PLA and provides an opportunity to determine whether the fibrous filler influences the morphology of the surface formed during the FFF process and the subsequent development of the electrolytically deposited Cu layer.

PLA-Brass is a composite material formed by a PLA matrix containing brass particles. This material was included to study a polymer substrate containing a metal filler and to compare its behaviour with both unfilled PLA and PLA-CF containing carbon reinforcement. Incorporating metallic fillers provides another material variable that can influence the character of the FFF surface and the subsequent formation of the Cu layer.

PETG (polyethylene terephthalate glycol-modified) represents a second unfilled polymer material, chemically and rheologically different from PLA. Inclusion of an alternative polymer serves to determine whether the changes observed during electrolytic copper plating are specific to PLA-based materials or if the nature of the polymer substrate also plays a significant role.

### 2.2. FFF Printing Strategy

All specimens were fabricated using a Bambu Lab A1 FFF (Bambo Lab, Shenzhen, China) equipped with a 0.4 mm nozzle and using 1.75 mm filament. The 0.20 mm Standard printing profile was used, with a layer height of 0.20 mm and an infill density of 15%. The top-surface printing speed was set to 200 mm·s^−1^. The nozzle temperature was set to 220 °C for all PLA-based materials (PLA, PLA-CF, and PLA-Brass), in accordance with the manufacturer’s recommendation, and to 255 °C for PETG.

For each experimental material, three variant samples were prepared, differing in printing strategy. Linear, circular and zigzag strategies were employed; the resulting 3D surface topographies are shown in Figure 1. The application of different printing strategies led to the formation of characteristic initial surface morphologies. This experimental design provides an opportunity to evaluate the influence of the substrate material and the surface morphology created by the FFF printing on the subsequent formation and final morphology of the electrodeposited Cu layer.

### 2.3. Conductive Layer Preparation and Electrolytic Copper Plating

The electrolytic copper plating procedure was based on the methodology used in our previous study [13]. The same deposition conditions were intentionally maintained in this work to evaluate the influence of the substrate material and the surface morphology created by FFF printing independently of changes in the electrolytic deposition process.

Before the electrolytic copper plating, the electrically non-conductive FFF substrates were coated with a conductive graphite interlayer. The samples were first cleaned with ethanol and dried. Subsequently, Graphite33 graphite spray (CRC Industries Europe, Zele, Belgium) containing finely dispersed graphite particles was applied to the entire surface. The graphite layer was left to cure for 4 h. The same graphite-layer preparation procedure was used for all specimens to minimise differences associated with the conductive interlayer. However, the thickness, electrical conductivity, surface morphology, spatial uniformity, and penetration of the graphite layer into surface valleys were not independently characterised in the present study. Therefore, possible local differences in the graphite interlayer between individual specimens cannot be excluded and may have contributed to the subsequent Cu deposition. This limitation should be considered when interpreting differences attributed to the FFF substrate material and initial surface topography.

Copper was electrolytically deposited from a commercial acidic electrolyte Pragogal Cu 2500 (Pragochema, Prague, Czech Republic) containing 50 g·L^−1^ Cu, 70 g·L^−1^ H_2_SO_4_, and 60 mg·L^−1^ Cl^−^. A 99.9% pure copper wire placed in a titanium basket was used as the soluble anode. Each specimen was electroplated individually and positioned vertically in the electrolyte. All specimens had identical dimensions and were immersed to the same depth; therefore, the exposed cathode area was the same for all experiments. The electrode arrangement and the specimen position relative to the anode were kept constant, with an anode–cathode distance of 6 cm. The electrodeposition was carried out at a current density of 1.9 A·dm^−2^ for 240 min at room temperature. The electrolyte was stirred with a magnetic stirrer at 50 rpm during deposition. Thus, the exposed cathode area, specimen orientation, electrode arrangement, anode–cathode distance, and electrochemical conditions were kept constant for all specimens. However, the local current-density distribution over the three-dimensional surface was not measured and may vary depending on the surface topography.

### 2.4. Measurement and Evaluation of Surface Topography

Surface topography was measured using an Olympus LEXT OLS5000 laser confocal microscope (Evident Corporation, Tokyo, Japan). The confocal optical system uses a semiconductor laser with a light source with wavelength 405 nm and a highly sensitive spectral detector (photomultiplier). The characteristic shallow depth of field of the confocal system allows precise optical resolution of surface details and acquisition of three-dimensional data for 3D topography reconstruction. This non-contact method was used to characterise the original surfaces created by FFF printing as well as surfaces after the electrolytic deposition of a Cu layer.

An area of approximately 5 × 5 mm was scanned for each sample. The 3D topography analysis was performed in MountainsSPIP^®^ Expert 11 software (Digital Surf, Besançon, France). The values of individual parameters are reported as the arithmetic mean ± standard deviation calculated from five evaluated areas.

The surface topography parameters were evaluated in accordance with the ISO 25178 series of standards [15]. The height parameters Sa, Sp, Sv, Sz and Sku, and the hybrid parameter Sdr were evaluated on the S-L surface using a 2.5 µm S-filter and a 2.5 mm L-filter. Areal roughness parameters are among the most used characteristics for the evaluation of engineering surface texture in mechanical engineering and other industrial applications. The S-L surface was therefore used for the standard evaluation of areal roughness. During its generation, the L-filter removes the large-scale surface components, thereby separating roughness from larger spatial features of the surface. This approach is suitable for comparing the areal roughness of individual surfaces; however, part of the information related to the larger-scale surface morphology is removed.

For the evaluation of the volume parameters Vmp and Vvv, the S-F surface was used. The S-F surface is derived from the primary surface by removal of form using an F-operation, but without subsequent application of an L-filter. In FFF surfaces, the large-scale peaks and valleys generated by the material deposition strategy represent an important part of the characteristic surface morphology. Their removal by L-filtering would lead to the loss of information relevant to the evaluation of the original FFF topography and its changes after Cu deposition. The S-F surface was therefore used for the volume analysis in order to preserve these spatial characteristics.

The parameter Sku (kurtosis) characterises the kurtosis of the surface height distribution. Sp (maximum peak height) represents the height of the highest peak above the mean plane, while Sv (maximum valley depth) indicates the depth of the deepest depression below the mean plane. The parameter Sz (maximum height) expresses the total height range of the surface as the sum of Sp and Sv. Sa (arithmetical mean height) represents the average absolute height deviation of the surface from the mean plane. The parameter Sdr (developed interfacial area ratio) expresses the percentage increase in the actual surface area compared to its projected area and thus provides additional information about the geometric structure of the surface. The evaluation was further supplemented by the volume parameters Vmp (peak material volume), which characterises the volume of material in the peak area, and Vvv (dale void volume), which characterises the volume of empty space in the deepest depressions of the surface.

The same measurement, processing and data evaluation procedure was used for surfaces before and after electrolytic Cu deposition. Direct comparison of both states therefore allows changes in surface morphology associated with Cu deposition to be quantified non-destructively.

To quantitatively compare the relative contributions of substrate material and printing strategy to the observed variability in surface topography, a descriptive factorial decomposition was performed for the selected height parameters Sa, Sp, Sv, and Sz. The analysis was carried out separately for the original and Cu-coated surfaces using the specimen-level mean values for the 12 Material × Strategy combinations (four materials × three printing strategies). The total variability among these mean values was decomposed into components associated with Material, Strategy, and the Material × Strategy term. Each component was expressed as a percentage of the total observed variability.

## 3. Results and Discussion

To assess the effect of the electrolytically deposited Cu layer, the surface topography before and after plating was compared. Different FFF printing strategies enabled monitoring the influence of the substrate material and its initial morphology on the resulting character of the plated surface. The analysis is based on a combination of selected surface and volume parameters that allow quantification of the changes in topography arising during the formation of the Cu layer.

The surface change is shown in Figure 2. A comparison of the original surface before plating (Figure 2a) and the surface after the electrolytic deposition of Cu (Figure 2b) shows that the Cu layer retains the basic morphology created by FFF printing, but at the same time changes its local character. A cross-section of the sample (Figure 2c) shows that some cavities of the original surface can be bridged by the coating. Therefore, the resulting surface of the Cu layer may no longer reproduce the deepest parts of the original topography. This observation demonstrates that the deposited Cu layer follows the basic geometry of the FFF substrate but is not an exact geometric copy of the original surface. Similar effects of the initial substrate morphology on the resulting electrodeposited Cu surface have been reported previously [6,10,16].

Consequently, the investigation focused not exclusively on the variation in the total amplitude of the topographical deviations, but also on changes in the height distribution, peak and valley regions, developed area, and volumetric characteristics of the surface.

The Sku parameter allows evaluation of the change in the kurtosis of the surface height distribution after Cu deposition. Subsequent evaluation of Sp and Sv provides complementary information on changes in the maximum peak height and maximum pit depth. Other parameters supplement this description with information on the total amplitude, developed area, and volumetric characteristics of the surface.

In Figure 3, a decrease in Sku is observed for all investigated configurations after Cu deposition. The decrease in Sku indicates a change in the kurtosis of the surface height distribution. The interpretation of Sku was therefore considered together with Sp and Sv rather than independently. The evaluation of the surface roughness height parameters Sa, Sp, Sv and Sz indicates that Cu deposition leads to characteristic changes in the amplitude properties of the original FFF topography. Simultaneously, the absolute level of the height parameters is strongly dependent on the printing strategy. The highest amplitudes are observed for zigzag surfaces, whereas linear and circular strategies generally produce less pronounced height differences. This distinction remains visible after Cu deposition, indicating that the resulting Cu surface retains the main morphological characteristics associated with the original FFF printing strategy.

The Sa parameter (Figure 4a) generally decreased after Cu deposition. More pronounced changes were observed for surfaces with higher initial Sa values. For example, Sa decreased from 42.97 ± 7.3 to 29.35 ± 5.8 µm for zigzag PETG, whereas only minor changes were observed for linear PETG, from 21.57 ± 2.4 to 21.36 ± 2.9 µm, and linear PLA-Brass, from 6.24 ± 0.5 to 6.27 ± 0.1 µm. Overall, Sa exhibited a tendency to decrease after Cu deposition, although the magnitude of the change depended on the initial surface morphology.

The Sp parameter (Figure 4b) showed a different trend. In contrast to Sa, the maximum peak height generally increased after Cu deposition. This trend was particularly pronounced for the linear strategy, with Sp increasing from 25.42 ± 1.0 to 32.20 ± 0.7 µm for PLA, from 43.73 ± 0.8 to 45.49 ± 2.3 µm for PLA-CF, from 44.64 ± 1.7 to 50.03 ± 1.5 µm for PLA-Brass, and from 58.54 ± 3.6 to 70.7 ± 12.9 µm for PETG. A similar increase was observed for most zigzag surfaces. These results indicate that the decrease in Sa cannot be attributed to uniform smoothing of the entire surface topography. Instead, the peak regions were preserved, and, in some cases, the maximum peak heights increased after Cu deposition.

The opposite tendency is evident for the parameter Sv (Figure 4c). For the majority of the analysed surfaces, the maximum depth of the depressions decreases after the formation of the Cu coating. This effect is particularly pronounced for the circular strategy, where Sv decreases for PLA from 92.05 ± 13.2 to 73.55 ± 7.9 µm, for PLA-CF from 94.02 ± 13.8 to 48.18 ± 5.9 µm, for PLA-Brass from 108.1 ± 7.9 to 44.39 ± 3.4 µm and for PETG from 173.2 ± 12.2 to 151.6 ± 11.9 µm. The reduction in Sv corresponds to a reduction in the maximum depth of the surface depressions. This result is consistent with the cross-sectional observations in Figure 2c, where local bridging of some surface depressions by the Cu layer is visible.

The relationship between Sp and Sv is crucial for the interpretation of Sz (Figure 4d). The parameter Sz represents the total height range of the surface, and therefore its change depends on the simultaneous development of both components. If the decrease in Sv is greater than the increase in Sp, Sz decreases after Cu deposition. This phenomenon is clearly illustrated, for instance, for circular PLA-Brass. Conversely, where the increase in Sp outweighs the change in Sv, Sz may remain similar or increase. Sz should therefore be interpreted in relation to Sp and Sv rather than independently as an indicator of surface smoothing or roughening.

The changes observed in Sp and Sv can be discussed in the context of geometry-dependent Cu electrodeposition. Preferential Cu deposition on protruding regions of cathodic surfaces has previously been related to differences in local current distribution and metal-ion supply [16]. Studies of Cu electrodeposition in three-dimensional recessed features further show that local deposition can be influenced by current localisation, ionic concentration gradients, electromigration, and convective transport [17,18,19]. Braun et al. demonstrated by numerical simulation that Cu deposition in microvias is affected by local ion depletion, electrolyte conductivity, electromigration and convection [17]. Moffat et al. described the importance of local geometry and mass transport during Cu growth in three-dimensional features [18], while Feng et al. experimentally observed non-uniform Cu deposition within microcavities under different transport conditions [19]. The morphology of electrodeposited Cu can also be affected by electrodeposition conditions and by the initial surface morphology of the substrate [20,21]. These studies provide a mechanistic framework for interpreting the present morphological observations. However, local current-density distribution and ion transport were not measured in the present study, and these mechanisms are therefore considered literature-supported interpretations rather than directly demonstrated mechanisms.

The Sdr parameter, graphically shown in Figure 5, provides additional information to the height parameters, as it characterizes the development of the actual surface area relative to its projected area. The results showed that the change in Sdr after the formation of the Cu coating is not uniform but strongly depends on the combination of the substrate material and the printing strategy. This trend is supported by the cross-sections of the Cu coatings (Figure 6), which show differences in the morphology of the Cu coating relative to the original topography among the individual materials. Especially for PETG, a more pronounced local variability of the geometry of the Cu coating is evident, which is in accordance with the high Sdr values achieved after its formation. For PLA-CF, the Sdr values before and after Cu deposition remained very similar for all strategies, indicating only limited changes in the developed interfacial area ratio. For PLA, a more pronounced decrease was recorded only for the linear strategy, while the circular and zigzag surfaces showed only small changes. Different behaviour was observed for PLA-Brass and especially PETG. For PLA-Brass with the zigzag strategy, Sdr increased from 16.20% to 58.89%, while it decreased for the linear and circular strategies. For PETG, Sdr increased for all three printing strategies, with the resulting Cu surface values reaching 73.08–80.30%. The relatively similar Sdr values obtained after Cu deposition despite the different initial topographies indicate that the formation of the Cu layer substantially modified the developed interfacial area ratio of the PETG surfaces.

Comparing Sdr with the height parameters shows that a decrease in Sa, Sv or Sz cannot automatically be interpreted as a decrease in the developed surface area. The PETG results are particularly important because the increase in Sdr occurred despite the generally lower Sa values after Cu deposition. This demonstrates that a reduction in height amplitude does not necessarily correspond to a reduction in the developed interfacial area ratio. Sdr therefore provides complementary geometric information to the height parameters but does not by itself identify the spatial scale or origin of the additional developed area.

The cross-sections in Figure 6 show that the Cu layer follows the basic morphology of the FFF substrate. At the same time, the outer surface of the Cu coating is not an exact geometric copy of the substrate–Cu interface. Some local depressions may be bridged so that their deepest regions are no longer fully reflected in the outer surface of the coating. This phenomenon is particularly evident for PETG. The observed morphology supports the results of the topographic analysis of the parameters Vmp and Vvv, which describe the peak material volume and dale void volume, respectively.

The Vmp parameter increased after Cu electrodeposition for all investigated materials and printing strategies (Figure 7), although the magnitude of this increase varied considerably among the individual surfaces. Only minor changes were observed for circular and zigzag PLA, with increases of 1.8% and 5.4%, respectively. In contrast, the most pronounced increases were found for linear PLA and linear PETG, where Vmp increased by 170.2% and 159.9%, respectively. Substantial increases were also observed for circular PETG (+95.0%) and zigzag PLA-Brass (+75.9%). Overall, Cu electrodeposition was associated with an increase in the material volume of the peak region for all investigated surfaces, while the magnitude of this change varied markedly depending on the material and initial surface topography.

Vvv shows exactly the opposite trend. As can be seen from Figure 8, the Vvv parameter decreases in all twelve configurations after the Cu coating is formed. A pronounced decrease is especially noticeable for the more structured zigzag surfaces. For PLA-Brass, Vvv decreased from 0.005525 to 0.002635 mm^3^/mm^2^, which represents a decrease of 52.3%. For PETG, there was a decrease from 0.023420 to 0.013480 mm^3^/mm^2^, which is a decrease of 42.4%, while for PLA-CF, the value decreased from 0.011280 to 0.009857 mm^3^/mm^2^, which corresponds to a decrease of 12.6%. A pronounced decrease was also observed for PETG with a circular strategy, where Vvv decreased from 0.007375 to 0.003711 mm^3^/mm^2^, which is a decrease of 49.7%. A decrease in Vvv means a reduction in the empty volume of the deepest valley parts of the surface.

The volume parameters therefore provide complementary information on the modification of the peak and valley regions. The simultaneous increase in Vmp and decrease in Vvv demonstrate a change in the volumetric characteristics of the original FFF topography after Cu deposition. Together with the cross-sectional observations, these results are consistent with modification of the peak and valley regions and local bridging of some depressions. However, Vmp and Vvv themselves do not identify the physical mechanism responsible for these changes.

Table 1 summarizes the percentage contribution of substrate material, printing strategy, and the Material × Strategy term to the total variability of the selected surface height parameters for the original and Cu-coated surfaces. For all evaluated parameters, printing strategy showed the largest contribution to the total variability both before and after Cu deposition. For the original surfaces, the contribution of printing strategy ranged from 56.83% for Sa to 69.65% for Sv, whereas the contribution of substrate material ranged from 22.27% to 35.40%. After Cu deposition, the contribution of printing strategy ranged from 56.86% to 64.51%, while the contribution of substrate material ranged from 23.10% to 37.44%. For Sp, the percentage contributions changed only slightly after Cu deposition. For Sv and Sz, the contribution of printing strategy decreased by 7.30 and 5.03 percentage points, respectively, while the contribution of substrate material increased by 3.82 and 3.73 percentage points. For Sa, the contribution of printing strategy remained nearly unchanged, from 56.83% to 56.86%, whereas the contribution of substrate material decreased from 30.79% to 23.10%. These results show that printing strategy accounts for a larger proportion of the observed variability in the selected height parameters than substrate material, both for the original and Cu-coated surfaces.

This result directly addresses the main objective of the study. Although the resultant surface is governed by a combination of the substrate material and the printing strategy, and the resulting surface topography, the descriptive factorial decomposition shows that the printing strategy accounts for the larger proportion of the observed variability in all four evaluated height parameters. Importantly, this predominance remains after Cu deposition. The result is consistent with the observation that the Cu layer retains the main morphological characteristics generated by the original FFF printing strategy.

From an application perspective, the present results demonstrate the usefulness of non-destructive 3D surface topography for quantifying the morphology of the resulting Cu-coated surface. The combined evaluation of Sku, Sp, Sv, Sa, Sz, Sdr, Vmp and Vvv describes complementary aspects of the height distribution, peak and valley regions, developed surface area and volumetric characteristics. Although these parameters define surface morphology, they do not directly reflect other functional properties such as coating adhesion, thickness, microstructure, or comprehensive quality. The present study also has several limitations. Local current-density distribution, ion transport, nucleation kinetics and Cu microstructure were not directly measured. Although all specimens were electroplated under the same macroscopic electrochemical and geometric conditions, the local current-density distribution may still depend on the three-dimensional surface geometry. In addition, although the same graphite spray and application procedure were used for all specimens, the thickness, electrical conductivity, surface morphology, spatial uniformity, and possible penetration of the graphite interlayer into surface valleys were not independently characterized. Therefore, a possible contribution of local variations in the conductive graphite interlayer to the resulting Cu morphology cannot be excluded. Furthermore, changes in Sdr, Vmp or Vvv cannot by themselves be interpreted as direct measures of adhesion or coating quality. Future studies should therefore combine non-destructive 3D topography with local coating-thickness measurements, scanning electron microscopy (SEM) characterisation of coating morphology and microstructure, adhesion testing, and subsequent functional or tribological evaluation.

## 4. Conclusions

This study investigated the relative influence of FFF substrate material and printing strategy on the surface topography of electrodeposited Cu layers under identical macroscopic electroplating conditions. The descriptive factorial decomposition showed that printing strategy accounted for a larger proportion of the observed variability in the selected height parameters than substrate material, both before and after Cu deposition. For the Cu-coated surfaces, the contribution associated with printing strategy ranged from 56.86% to 64.51%, whereas the contribution associated with substrate material ranged from 23.10% to 37.44%. These results indicate that the initial surface morphology generated by the FFF printing strategy remains an important factor in determining the resulting Cu surface topography.

The Cu-coated surfaces retained the main morphological characteristics of the original FFF surfaces but were not exact geometric copies of them. Sku decreased in all investigated configurations, indicating a change in the kurtosis of the surface height distribution. Sa generally decreased, Sv decreased for most surfaces, whereas Sp increased in most cases. Vmp increased in all twelve material–strategy combinations, while Vvv decreased in all cases. These changes demonstrate modifications in the height and volumetric characteristics of the peak and valley regions after Cu deposition. Cross-sectional observations additionally showed local bridging of some surface depressions. The topographic parameters themselves, however, do not identify the physical mechanism responsible for these changes.

The Sdr results further showed that changes in height amplitude do not necessarily correspond to changes in developed surface area. This was particularly evident for PETG, for which Sdr reached approximately 73–80% after Cu deposition for all three printing strategies. The combined evaluation of height, hybrid and volume parameters therefore provides complementary information on different aspects of the resulting Cu surface morphology.

Non-destructive 3D surface topography was shown to detect and quantify morphological differences between the original and Cu-coated surfaces. Its use in the present study is limited to surface morphology characterisation and does not establish coating thickness, adhesion, microstructure, homogeneity or functional performance. Further studies combining 3D topography with coating-thickness measurements, SEM characterization, adhesion testing and functional evaluation are required before topographic parameters can be validated as non-destructive indicators of Cu coating quality.

## Figures and Tables

**Figure 1:**
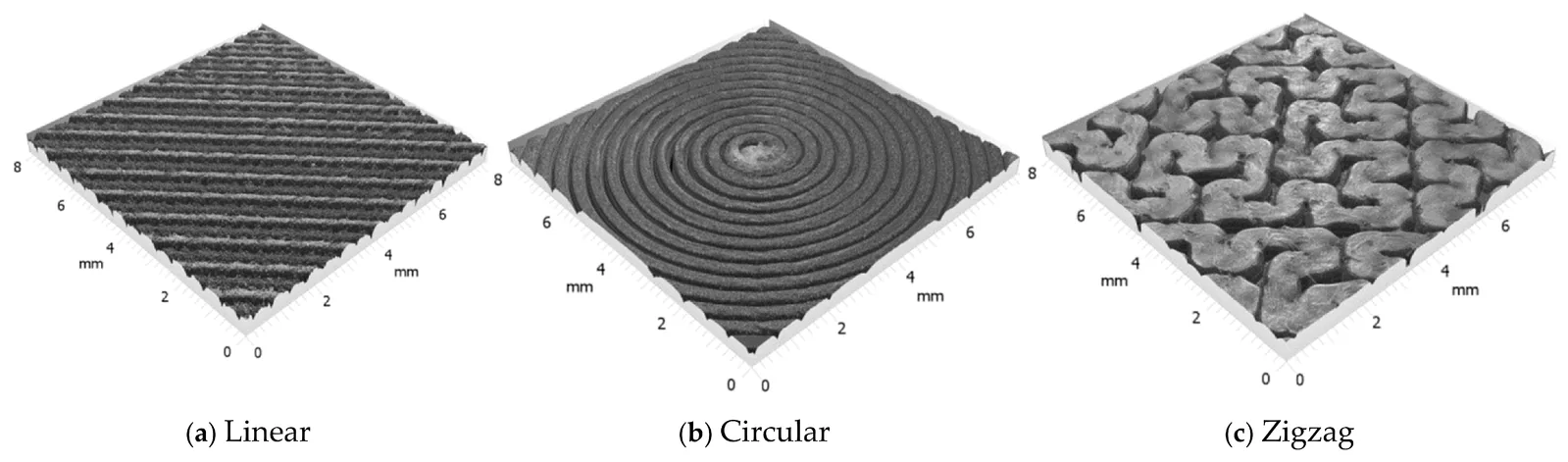
Representative 3D surface topographies generated using different FFF printing strategies: (**a**) Linear, (**b**) Circular, and (**c**) Zigzag.

**Figure 2:**
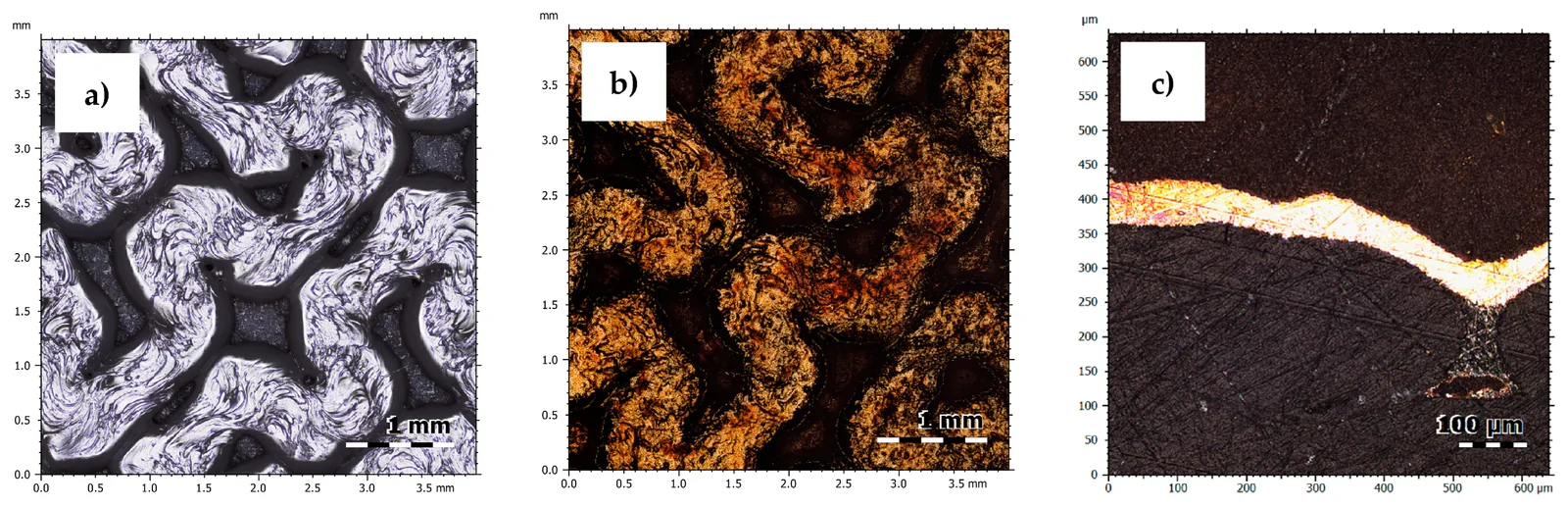
Image of the FFF surface and the electrodeposited Cu layer: (**a**) original surface before Cu deposition, (**b**) surface after Cu electrodeposition, and (**c**) cross-section of the sample showing the Cu layer on the polymer substrate.

**Figure 3:**
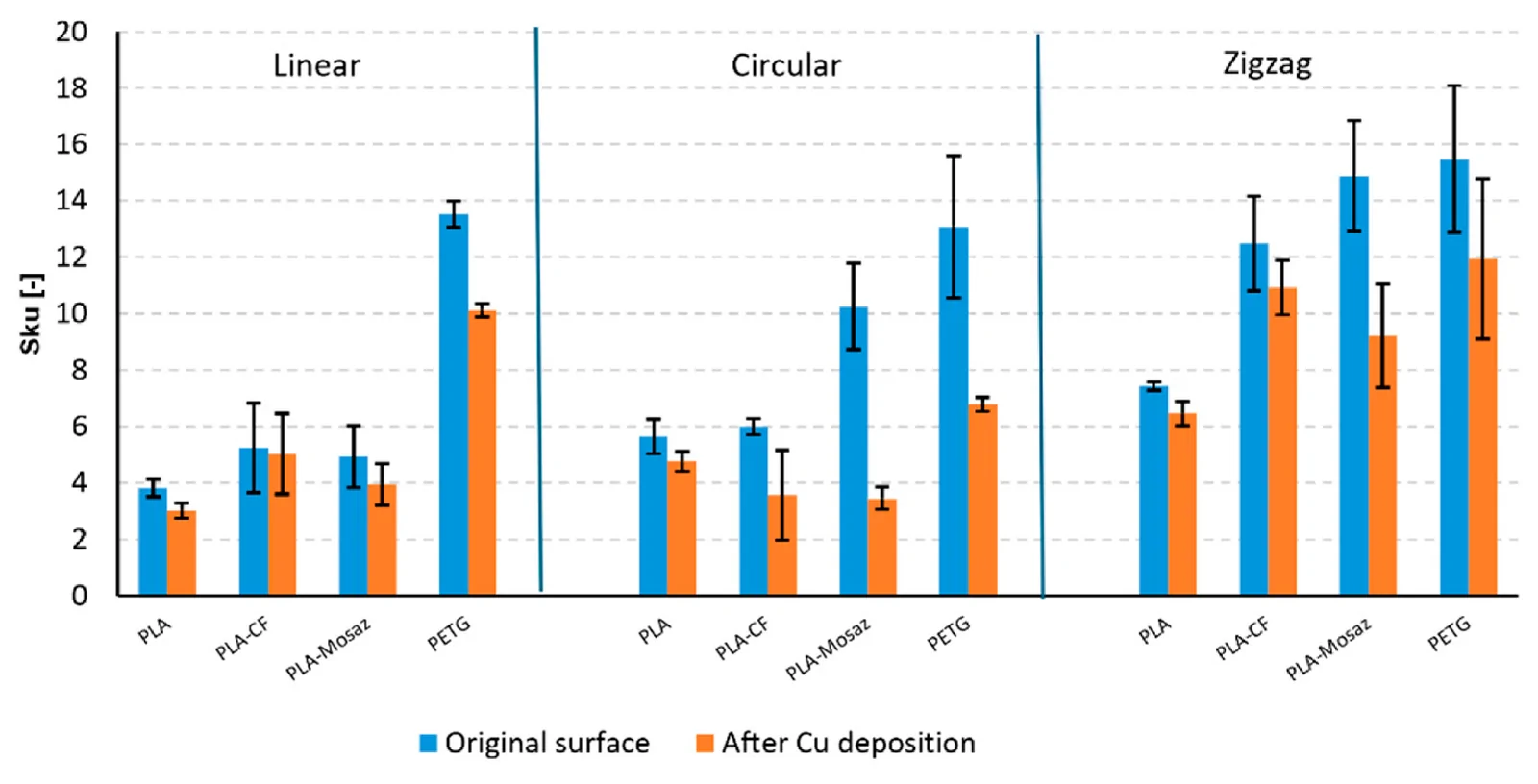
Comparison of the Sku parameter before and after Cu electrodeposition for different materials and FFF printing strategies.

**Figure 4:**
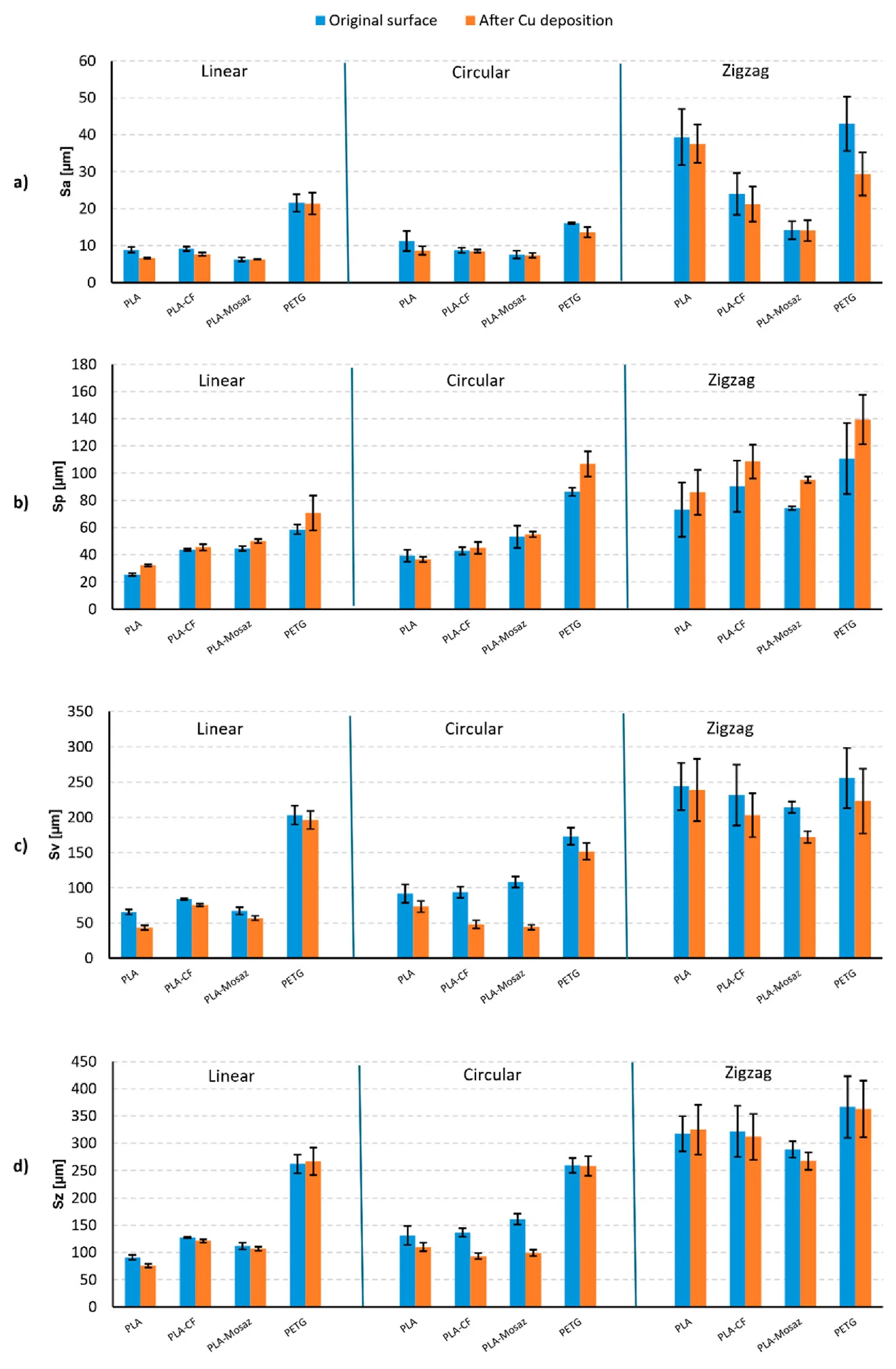
Comparison of the areal height parameters of the original FFF surfaces and the surfaces after Cu deposition for different materials and printing strategies: (**a**) arithmetic mean height, Sa; (**b**) maximum peak height, Sp; (**c**) maximum pit height, Sv; and (**d**) maximum height, Sz.

**Figure 5:**
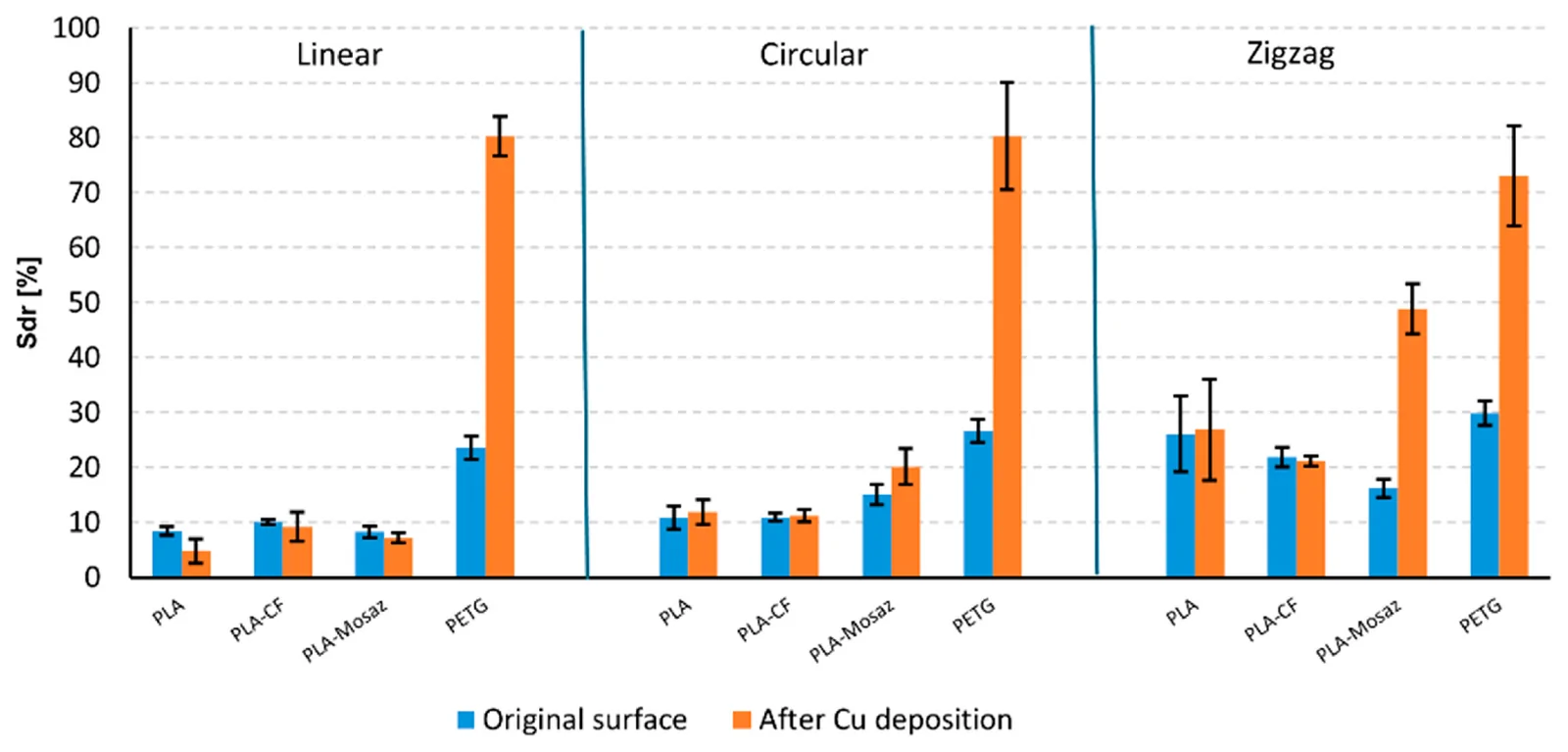
Comparison of the Sdr parameter of the original FFF surfaces and the surfaces after Cu deposition for different materials and printing strategies.

**Figure 6:**
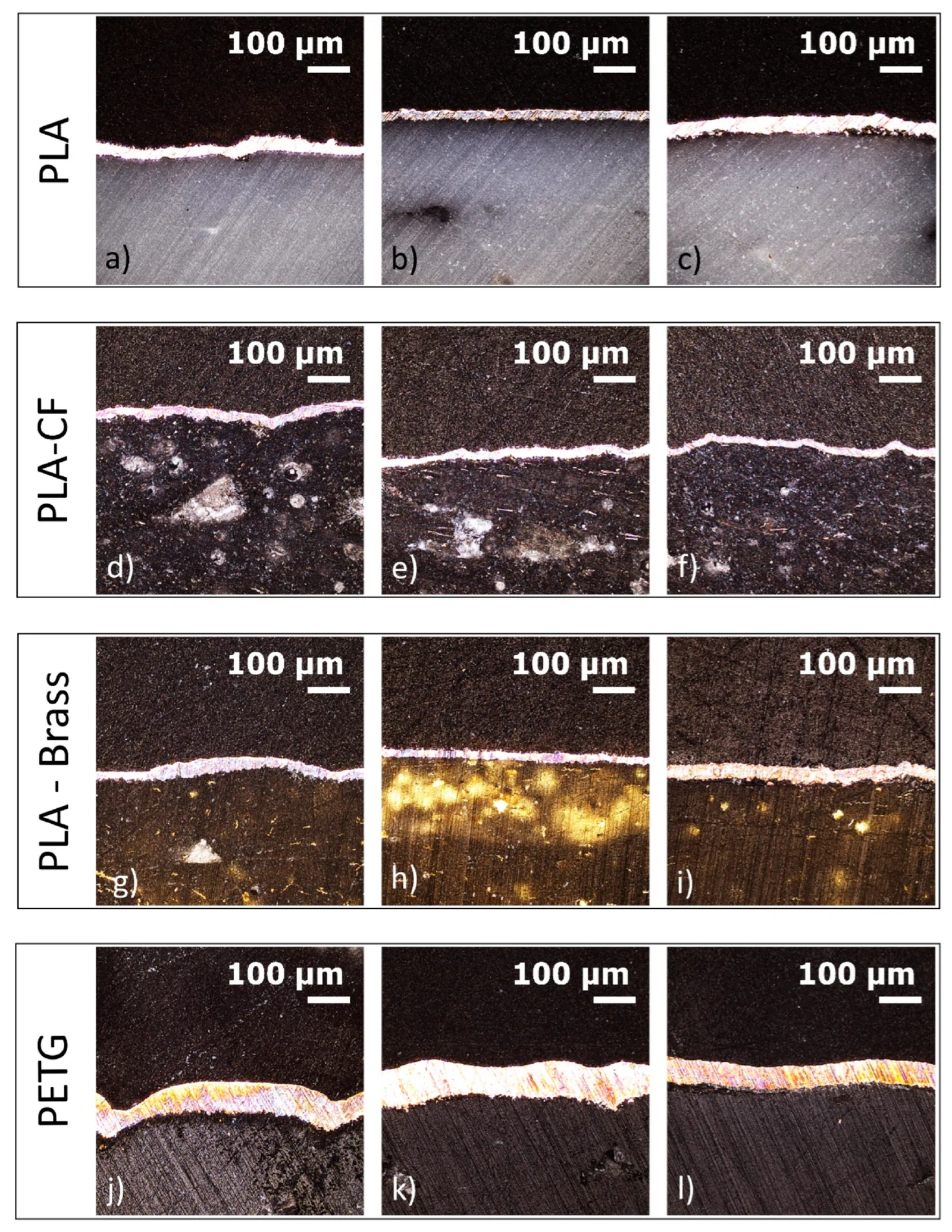
Cross-sections of FFF samples after electrodeposition of the Cu coating for the individual materials and printing strategies: (**a**–**c**) PLA, (**d**–**f**) PLA-CF, (**g**–**i**) PLA-Brass, and (**j**–**l**) PETG. From left to right, the linear, circular, and zigzag printing strategies are shown.

**Figure 7:**
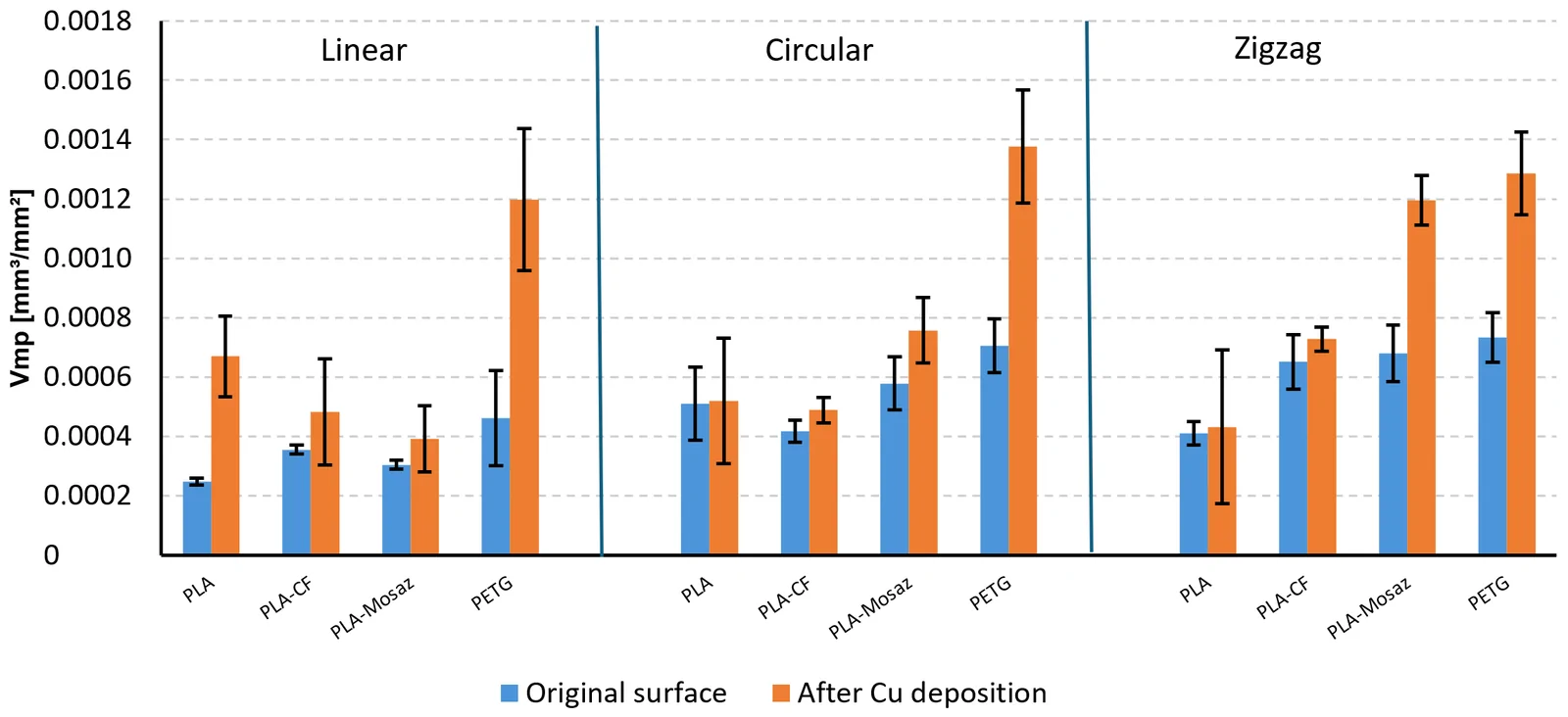
Comparison of the Vmp parameter of the original FFF surfaces and the surfaces after Cu deposition for PLA, PLA-CF, PLA-Brass, and PETG with linear, circular, and zigzag printing strategies.

**Figure 8:**
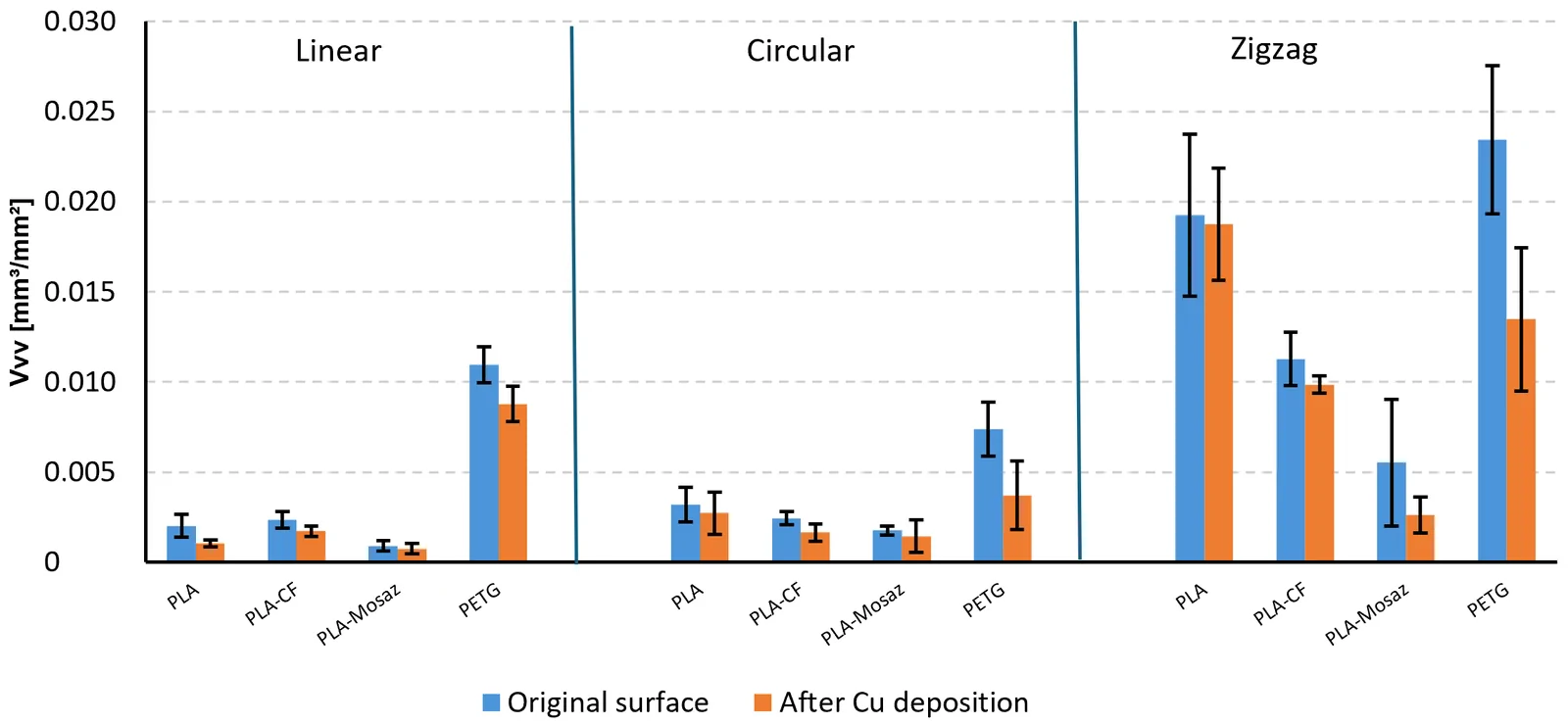
Comparison of the Vvv parameter of the original FFF surfaces and the surfaces after Cu deposition for PLA, PLA-CF, PLA-Brass, and PETG with linear, circular, and zigzag printing strategies.

**Table 1:** Factorial decomposition of variability in selected surface topography parameters before and after Cu deposition.

	Parameter	Material (%)	Strategy (%)	Material × Strategy Component (%)
Original surface	Sa	30.79	56.83	12.38
	Sp	35.40	59.12	5.48
	Sv	22.27	69.65	8.08
	Sz	25.76	69.54	4.70
After Cu deposition	Sa	23.10	56.86	20.04
	Sp	37.44	58.17	4.39
	Sv	26.10	62.34	11.56
	Sz	29.49	64.51	6.00

## Data Availability

The original contributions presented in this study are included in the article. Further inquiries can be directed to the corresponding author.
